# Acute hemolysis and methemoglobinemia secondary to fava beans ingestion in a patient with G6PD deficiency: A case report of a rare co-occurrence

**DOI:** 10.1097/MD.0000000000027904

**Published:** 2021-11-24

**Authors:** Husam Al-Dubai, Abdulrahman Al-Mashdali, Yousef Hailan

**Affiliations:** Internal Medicine Department, Hamad General Hospital, HMC, Doha, Qatar.

**Keywords:** favism, glucose-6-phosphate dehydrogenase deficiency, hemolysis, methemoglobinemia

## Abstract

**Rationale:**

Favism is a well-known cause of acute hemolytic anemia. Rarely, methemoglobinemia can also happen because of fava bean ingestion in patients with glucose-6-phosphate dehydrogenase (G6PD) deficiency. Few cases with this co-occurrence have been reported in the literature.

**Patient concerns:**

We report a case of a 47-year-old patient who presented with jaundice that started 2 days after eating fava beans.

**Diagnoses:**

Laboratory investigations revealed anemia with evidence of hemolysis (high reticulocytes count, high indirect bilirubin, bite cells in peripheral smear). Blood gases showed high methemoglobin level. Reduced level of G6PD enzyme confirmed the diagnosis of G6PD deficiency.

**Intervention:**

The patient was kept on supplemental oxygen. He was counselled to avoid food and drugs that can cause acute hemolysis.

**Outcomes:**

Oxygen saturation improved gradually. The patient was discharged without any complications after 2 days.

**Lessons:**

Patients with G6PD deficiency can develop both acute hemolytic anemia and methemoglobinemia secondary to fava beans ingestion. These patients should not receive methylene blue to avoid worsening hemolysis.

## 1 Introduction

Glucose-6-phosphate dehydrogenase (G6PD) deficiency is a common cause of hemolytic anemia with approximately 400 million affected people globally.^[1]^ Patients with G6PD deficiency are typically asymptomatic unless they become exposed to an oxidative stress which induces acute hemolysis. Fava beans ingestion is a well-known factor that can lead to acute hemolysis. Other triggers include infections and certain drugs.^[2]^

Methemoglobin (MetHb) is an abnormal oxidized form of hemoglobin (Hb) in which the heme iron configuration is changed from ferrous (Fe^2+^) to ferric (Fe^3+^) state. Methemoglobinemia is most of the time acquired, resulting from exposure to oxidizing agents such as medications^[3]^ and chemicals. Rarely, methemoglobinemia can be congenital.^[4]^

In patients with G6PD deficiency, acute hemolysis and methemoglobinemia can happen due to exposure to fava beans. To the best of our knowledge, this co-occurrence is very uncommon and has been described in the literature infrequently. Herein, we report a 47-year-old patient who developed this seldom condition.

## 2 Case presentation

The patient is a 47-year-old gentleman with a past medical history of hypertension and type-2 diabetes mellitus. He presented to the emergency department with a 3-day history of yellowish discoloration of his eyes and red urine. He denied any history of fever or abdominal pain. He did not complain from dyspnea or dizziness. He is complaint to his home medications and did not start any new medicine recently. He recalled eating a medium-sized plate of fava beans 2 days prior to his symptoms.

In the emergency department, he was febrile 38.5°C with blood pressure of 125/78 mm Hg. His heart rate was 117 beats per minute and respiratory rate was 20 breaths per minute. Oxygen saturation (SatO_2_) was 88% on room air. Upon examination, he had jaundice. He did not show any sign of distress. The rest of examination was unremarkable. He was put on supplemental oxygen and SatO_2_ barely increased to 90% to 91%. Blood gases analysis revealed SatO_2_ of 99% and MetHb level of 3.6% (Table 1).

**Table 1:** Laboratory results.

Parameter	Value	Reference range
WBC	17.3 × 10^3^/uL	4.0–10.0
Hgb	12 g/dL	13.0–17.0
Platelet	194 × 10^3^/uL	150–400
Reticulocytes	647.1 × 10^3^/uL (17.8%)	50–100 (0.5–2.5)
Bilirubin (total)	83.7	3.4–20.5
Bilirubin (direct)	11.1 μmol/L	0–8.6
MetHb	3.6%	0–1.5
G6PD	24 mU/10^9^ RBC	191–327

G6PD = glucose-6-phosphate dehydrogenase, Hgb = hemoglobin, MetHb = methemoglobin, WBC = white blood cell.

Complete blood count showed Hb of 12 g/dL (baseline 1 month before was 14.9 g/dL) with high reticulocytes count. Blood chemistry revealed hyperbilirubinemia which was mainly due to high indirect bilirubin (Table 1). We could not get haptoglobin and lactate dehydrogenase levels because the blood sample was hemolyzed. Peripheral smear showed mild normocytic normochromic anemia with marked reticulocytosis, some bite cells, and some nucleated red blood cells. Based on the history of recent ingestion of fava beans and the laboratory findings that are suggestive of acute hemolysis, G6PD deficiency was suspected. A quantitative G6PD assay was done, and the level was low, which confirmed the diagnosis of G6PD deficiency (Table 1).

The patient received paracetamol for the fever, which resolved within 24 hours. He was kept on supplemental oxygen and his SatO2 improved gradually. He did not require blood transfusion and became asymptomatic within 2 days. He was discharged after being counselled about the importance of avoiding food and drugs that may trigger hemolysis. A repeat Hb was done approximately 2 months after discharge, and it was within normal range.

## 3 Discussion

G6PD is an important enzyme for red blood cell integrity. It catalyzes the initial step in hexose monophosphate shunt that protects red blood cells against oxidative injury.^[5]^ G6PD-deficient red blood cells become susceptible to destruction by reticuloendothelial system after being exposed to an oxidant stress like fava bean. After ingestion of fava bean, reactive oxygen species are produced, which causes acute intravascular and extravascular hemolysis.^[6]^ Some individuals with G6PD deficiency develop mild and self-limiting hemolysis. On the other hand, severe and life-threatening hemolysis may occur in others.^[7]^ Once acute hemolytic episode happens, any inciting agent should be removed. Patients who become severely anemic require blood transfusion.

In normal Hb, iron is in the ferrous (Fe^2+^) state, which allows for binding and transporting of oxygen to the tissues. When the iron is oxidized, it changes to the ferric (Fe^3+^) state, forming MetHb. This abnormal form of Hb is incapable of oxygen transport, which reduces oxygen delivery to the tissues and increase the risk of tissue hypoxia.^[8]^ Physiologically, formation of MetHb is counteracted by various enzymes systems that reduce the iron back to the ferrous (Fe^2+^) state and keep MetHb level below 1%. One pathway involves nicotinamide adenine dinucleotide phosphate (NADPH)-MetHb reductase and uses NADPH as a cofactor. Production of NADPH is decreased in patients with G6PD deficiency. Consequently, this may lead to an oxidative state in the body, which increases the level of MetHb.^[8,9]^

Favism causing acute hemolysis and methemoglobinemia is very rare. Based on a recent literature review, 8 cases of this co-occurrence have been reported.^[9]^ All 8 patients were male with median age of 18 years (1–56) and MetHb level ranging from 3.5% to 35%. Among these cases, 1 patient received methylene blue before knowing that he is G6PD deficient, which resulted in worsening hemolysis.^[10]^ Two other patients were treated with vitamin C only.^[11,12]^ In our case, the level of MetHb was mildly elevated and the patient was asymptomatic and was not cyanosed. Therefore, we managed him conservatively until he recovered completely.

## 4 Conclusion

Although it is rare, acute hemolysis and methemoglobinemia can co-occur after fava bean ingestion in patients with G6PD deficiency. Patients with even mildly increased levels of methemoglobinemia may have significant hypoxia and may require hospital admission. Identifying the presence of this co-occurrence is vital as giving methylene blue can worsen the hemolysis.

## Acknowledgments

We would like to thank internal medicine residency program in Hamad Medical Corporation for the scientific support. Open Access funding was provided by the Qatar National Library.

## Author contributions

All authors contributed in writing and editing the manuscript.

**Conceptualization:** Husam Al-Dubai.

**Writing – original draft:** Husam Al-Dubai, Abdulrahman Al-Mashdali, Yousef Hailan.

**Writing – review & editing:** Husam Al-Dubai, Abdulrahman Al-Mashdali, Yousef Hailan.
